# Supplementary material for: Performance of a postnatal metabolic gestational age algorithm: a retrospective validation study among ethnic subgroups in Canada
Source: BMJ Open. 2017 Sep 3;7(9):e015615. doi: 10.1136/bmjopen-2016-015615 (PMC5589017; doi:10.1136/bmjopen-2016-015615)
Supplement: Author's manuscript [file bmjopen-2016-015615.draft_revisions.pdf]

BMJ Open

BMJ Open is committed to open peer review. As part of this commitment we make the peer review history of every article we publish publicly available.

When an article is published we post the peer reviewers' comments and the authors' responses online. We also post the versions of the paper that were used during peer review. These are the versions that the peer review comments apply to.

The versions of the paper that follow are the versions that were submitted during the peer review process. They are not the versions of record or the final published versions. They should not be cited or distributed as the published version of this manuscript.

BMJ Open is an open access journal and the full, final, typeset and author-corrected version of record of the manuscript is available on our site with no access controls, subscription charges or pay-per-view fees (http://bmjopen.bmj.com).

If you have any questions on BMJ Open's open peer review process please email editorial.bmjopen@bmj.com

BMJ Open

Validation of a gestational age prediction algorithm in ethnic subgroups using routinely collected newborn screening metabolic profiles

Journal:	*BMJ Open*
Manuscript ID	bmjopen-2016-015615
Article Type:	Research
Date Submitted by the Author:	20-Dec-2016

Complete List of Authors:	Hawken, Steven; Institute for Clinical Evaluative Sciences, ICES @uOttawa; The Ottawa Hospital Research Institute,<br>Ducharme, Robin; Institute for Clinical Evaluative Sciences<br>Murphy, Malia; Ottawa Hospital Research Institute<br>Atkinson, Katherine; Ottawa Hospital Research Institute<br>Potter, Beth; University of Ottawa, Epidemiology and Community Medicine<br>Chakraborty, Pranesh; Newborn Screening Ontario, Children's Hospital of Eastern Ontario<br>Wilson, Kumanan; Ottawa Hospital Research Institute, Clinical Epidemiology

**Primary Subject Heading**:	Obstetrics and gynaecology
Secondary Subject Heading:	Qualitative research
Keywords:	gestational age, prediction modelling, newborn screening

SCHOLARONE™ Manuscripts

Title: **Validation of a gestational age prediction algorithm in ethnic subgroups using routinely collected newborn screening metabolic profiles**

Authors: Steven Hawken[a,b,c], Robin Ducharme[c], Malia SQ Murphy[a], Katherine M Atkinson[a,d], Beth K Potter[a,b,c], Pranesh Chakraborty[e,f], Kumanan Wilson[a,b,c,g].

Affiliations: [a]Clinical Epidemiology Program, Ottawa Hospital Research Institute, Ottawa, Ontario, Canada
[b]School of Epidemiology, Public Health and Preventative Medicine, University of Ottawa, Ottawa, Ontario, Canada
[c]Institute for Clinical Evaluative Sciences (ICES uOttawa), Ottawa, Ontario, Canada
[d]Department of Public Health Sciences, Karolinska Institute, Stockholm, Sweden
[e]Department of Pediatrics, University of Ottawa, Ottawa, Ontario, Canada;
[f]Newborn Screening Ontario, Children's Hospital of Eastern Ontario, Ottawa, Ontario, Canada
[g]Department of Medicine, University of Ottawa, Ottawa, Ontario, Canada.

Corresponding Author: Kumanan Wilson, MD, MSc, FRCPC
Ottawa Hospital, Civic Campus
1053 Carling Avenue, Box 684
Administrative Services Building
Ottawa, ON K1Y 4E9
kwilson@ohri.ca
613-798-5555 ex 17921

Word Count: **1525**

Data Sharing Statement: All data used in this study were obtained from the Institute for Clinical Evaluative Sciences and are accessible to individuals with appropriate authorization.

For peer review only

ABSTRACT

Objectives: We and others have recently developed gestational age prediction algorithms derived from routinely collected newborn screening profiles. Validation of algorithm performance across ethnic subgroups is essential to determining potential utility in large multi-ethnic populations. We sought to determine the validity of such an algorithm among recent immigrants to Canada.

Design: We conducted a retrospective validation study in infants born in Ontario between April 2009 and September 2011.

Setting: Data were collected from the Institute for Clinical Evaluative Science at the Ottawa Hospital in Ontario, Canada.

Participants: The dataset included 230,034 infants born to non-landed-immigrants and 70, 098 infants born to immigrant mothers. The 5 most common countries of maternal origin were India (n=10,038), China (n=7,468), Pakistan (n=5,824), The Philippines (n=5,441) and Vietnam (n=1,408). Maternal country of origin was obtained from the Citizenship and Immigration Canada Landed Immigrant Database and used as a surrogate marker of infant ethnicity.

Primary and Secondary Outcome Measures: Algorithm predictive performance was evaluated across non-immigrant and immigrant populations.

Results: RMSE of 1.05 was observed for infants born to non-immigrant mothers, whereas RMSE ranged from 0.98–1.15 amongst infants born to immigrant mothers. AUC for distinguishing term vs preterm infants (≥37 vs <37 weeks gestational age or >34 vs ≤34 weeks gestational age) were 0.958 and 0.986 respectively in the non-immigrant subgroup and ranged from 0.927-0.964 and 0.966-0.99 in immigrant subgroups.

For peer review only

For peer review only - http://bmjopen.bmj.com/site/about/guidelines.xhtml

Conclusions: Algorithms for postnatal determination of gestational age may be further refined by development and validation of region or ethnicity-specific models however our results provide reassurance that our algorithm developed from Ontario datasets performs well across a range of ethnicities and countries of origin without modification.

Key Words: gestational age; prediction modelling; newborn screening

Strengths and Limitations of this Study

- Large sample size: the validation cohort included 300,132 live-born infants born between April 2009 and September 2011 who underwent newborn screening at Newborn Screening Ontario;
- Naturalized immigrants living in North America are not necessarily representative of individuals living in the country of origin, either due to admixture or environmental factors.

For peer review only

INTRODUCTION

Through their Grand Challenges Exploration program, The Bill and Melinda Gates Foundation have made new approaches to measuring brain function and development a key funding priority. Accurate estimation of gestational age at the time of birth is vital for the assessment of neurocognitive function and tracking developmental milestones.(1, 2) For some pregnancies, where antenatal ultrasound screening is not available, such as those occurring in jurisdictions with challenging socioeconomic conditions and/or limited access due to rurality, determination of gestational age can be challenging or impossible. The inability to determine preterm birth status impedes adequate provision of of newborn care. In these settings with limited access to ultrasound dating, newborn blood spots collected within the first few days of an infants life may offer a unique opportunity to assess gestational age at birth.

Newborn blood spots collected within the first few days of an infant's life may offer a unique opportunity to assess gestational age. We and others have recently demonstrated the accuracy of prediction algorithms derived from routinely collected newborn screening metabolic profiles for postnatal gestational age estimation in three independent North American cohorts.[3-5] However, metabolic profiles, and thus accuracy of such prediction models, may be subject to biological variation as a result of *in utero* environmental exposure or genetic influences. As the aforemention prediction models, including our own were originally developed using largely white populations, examining the robustness of our algorithm across a range of ethnic, geographical and socioeconomic conditions will be essential to determining if our algorithm can be applied in international low-resource settings. This study sought to determine the validity of a

For peer review only - http://bmjopen.bmj.com/site/about/guidelines.xhtml

postnatal gestational age prediction algorithm in ethnic subpopulations within Ontario, Canada.

MATERIALS AND METHODS

Study Design

We conducted a population-based retrospective validation study of infants born in the province of Ontario, using a combination of linked health administrative, newborn screening and immigration data maintained by the Institute for Clinical Evaluative Sciences (ICES). The Newborn Screening Ontario database includes the screening analyte profile of each infant who completed newborn screening (>99% of all infants born in Ontario). These screening analyte levels are used to screen for 29 conditions using a panel of over 40 screening analytes including acylcarnitines, amino acids, endocrine markers, enzymes and co-enzyme markers among others (Table 1). Maternal country of origin was used as a surrogate marker of infant ethnicity and was ascertained from the Citizenship and Immigration Canada Landed Immigrant Database. This study was approved by the Ottawa Health Science Network Research Ethics Board, Ottawa, Canada (20140724-01H).

For peer review only

Table 1. Newborn Screening Analytes used in model development

**Acylcarnitines**	C0, C2, C3, C3DC, C4, C4DC, C4OH, C5, C5:1, C5OH, C5DC, C6, C6DC, C8, C8:1, C10, C10:1, C12, C12:1, C14, C14:1, C14:2, C16, C16OH, C16:1OH, C18, C18:1, C18:2, C18OH, C18:1OH**,**
**Amino Acids**	alanine, arginine, citruline, glycine, leucine, methionine, ornithine, phenylalanine, tyrosine, valine,
**Endocrine Markers**	17-hydroxyprogesterone, thyroid stimulating hormone
**Enzyme and Co-Enzyme Markers**	galactose-1 phosphate uridyltransferase, biotinidase

Original Model

The original gestational age prediction model included 249, 700 infants who were born between April 2007 and March 2009. The details of the original model have been described previously.[3] Briefly, infants who were identified as positive for any disorder screened for by newborn screening Ontario were excluded, as were infants with missing or unsatisfactory samples, gestational age and birthweight. The data were randomly partitioned into model development (50%), validation (25%) and test (25%) subsamples. Multiple linear regression was performed in the model development set, in which all analyte main effects were included in the model, as well as birthweight and sex. For analytes and birthweight, squared and cubic terms were also included. A stepwise variable selection algorithm was then conducted and all pairwise interactions were considered for inclusion. The *Schwartz Bayesian Criterion*, which rewards improved model fit and penalizes model complexity, was used in variable selection. Once this process was complete, the mean square error for the fitted model at each step was calculated in the independent validation sample subset, and the model with the smallest mean square error was selected. This approach provides a high level of protection from overfitting in the final model.[3] Parameter estimates for the fitted model were fixed, and used to score (*i.e.* predict gestational age) in the independent test sample subset (n=62,434), and model performance characteristics (r-square, mean square error, proportion with observed versus predicted gestatinal age within 1 week) were calculated using this data, which was not used in model development. The final model included 43

For peer review only - http://bmjopen.bmj.com/site/about/guidelines.xhtml

effects, including infant birthweight, sex and newborn screening analyes and a total of 311 model terms.

Gestational age was then dichotomized to distinguish term or preterm infant subgroups. Model performance to correctly classify infants across two thresholds of preterm birth categories by logistic regression analysis was assessed (area under the receiver operator curve, sensitivity, specificity and positive predictive value). Preterm birth thresholds were <37 weeks gestational age, the clinical threshold for preterm birth, and ≤34 weeks gestational age, a threshold that represents the lower limit of the late preterm period. Infants born at >34 weeks gestation have reduced health risks compared to younger gestational age infants, while still at increased risk relative to infants born at term. Predicted gestational age identified by multiple linear regression was used as a continuous independent variable in logistic regressions predict the probability of being premature.[3]

For peer review only

Validation Cohort

The validation cohort included 300,132 live-born infants born between April 2009 and September 2011 who underwent newborn screening at Newborn Screening Ontario. The same exclusion criteria were applied as in the original study. The validation cohort was completely independent from the data used in the development of the original model.

Model Validation

Employing the multivariable linear regression model coefficients derived in our original study, continuous gestational ages for infants in the validation cohort were

For peer review only - http://bmjopen.bmj.com/site/about/guidelines.xhtml

predicted based on birthweight, sex and analyte levels. Prematurity status for infants based on dichotomous thresholds were also predicted using logistic regression as described above. Corresponding model performance characteristics were calculated in landed immigrant subgroups as well as in non-immigrants.

RESULTS

The validation dataset included 230, 034 infants born to non-immigrant mothers and 70, 098 to immigrant mothers. The 5 most common countries of maternal birth were India (n=10,038), China (n=7,468), Pakistan (n=5,824), the Philippines (n=5,441) and Vietnam (n=1,408). The most common countries from North and Sub-Saharan Africa were Somalia (n=833) and Nigeria (n=800), respectively.

Model performance characteristics for non-immigrant populations and the top eight landed immigrant subgroups are presented in Table 2. Original model performance characteristics are provided for comparison. Among non-immigrants, our algorithm performed comparably to our original validation. with areas under the receiver operator curve (AUC) for classifying infants as term versus preterm; ≥37 versus <37 weeks gestational age, AUC=0.96 and >34 versus ≤34 weeks gestational age, AUC=0.99. Absolute prediction was accurate to within ±1 week in continuous linear regression models, RMSE=1.05.

For peer review only - http://bmjopen.bmj.com/site/about/guidelines.xhtml

For peer review only

Table 2: Model Performance comparison for Original Validation Study and new validation in immigrant and non-immigrant subgroups.

Cohort	N total	RMSE	Infants with GA correctly classified		<37 weeks GA			≤34 weeks GA		
			±1 wks (%)	±2 wks (%)	N	AUC (95%CI)	p-value[a]	N	AUC (95%CI)	p-value[a]
Original Validation	62,434	1.06	66.8	94.9	3,844	0.970 (0.966,0.974)	-	1263	0.991 (0.987,0.995)	-
**Ethnic validation based on immigration status in IRCC data**										
Non-immigrants	230,034	1.05	67.0	94.9	17,033	0.958 (0.956,0.960)	<0.0001****	5,827	0.986 (0.988,0.992)	0.021*
India	10,038	1.04	68.1	95.2	681	0.947 (0.935,0.959)	0.00027***	244	0.984 (0.973,0.995)	0.240
China	7,468	0.98	71	96.2	362	0.957 (0.942,0.972)	0.092	113	0.989 (0.975,1.00)	0.78
Pakistan	5,824	1.05	67.2	95.3	377	0.949 (0.933,0.965)	0.010**	119	0.974 (0.954,0.994)	0.103
Philippines	5,441	1.05	66.5	94.7	451	0.958 (0.945,0.971)	0.082	178	0.994 (0.986,1.00)	0.505
Vietnam	1,408	1	70	95.2	86	0.949 (0.916,0.982)	0.209	27	0.988 (0.959,1.00)	0.840
Bangladesh	1,182	1.14	67.4	94.6	85	0.945 (0.911,0.979)	0.151	28	0.966 (0.919,1.00)	0.300
Somalia	833	1.15	62.1	93.6	58	0.927 (0.880,0.974)	0.072	19	0.991 (0.961,1.00)	1
Nigeria	800	1.08	65.1	94.6	56	0.964 (0.930,0.998)	0.733	21	0.966 (0.912,1.00)	0.369

For peer review only - http://bmjopen.bmj.com/site/about/guidelines.xhtml

RMSE, root mean squared error; wks, weeks; GA, gesetational age; AUC, area under the ROC curve; IRCC, Citizenship and Immigration Canada's Permanent Resident Database. [a]p-value for two-sided z-test for the difference between original and subgroup AUC.[6] *P≤0.05; **P≤0.01; ***P≤0.001; ****P≤0.0001

For peer review only

For peer review only - http://bmjopen.bmj.com/site/about/guidelines.xhtml

The prediction model was able to discriminate between dichotomous preterm birth categories of immigrant infants with excellent precision. For discrimination of ≥37 versus <37 weeks gestation, AUC ranged from a 0.927 amongst infants of maternal Somalian descent to 0.964 amongst infants of maternal Nigerian heritage. Similarly, the model was able to discriminate well between >34 weeks versus ≤34 weeks gestational age, with AUC ranging from 0.966 for Nigerian and Bangladeshi infants to 0.994 amongst phillipino infants. By linear regressional analysis RMSE ranged from 0.98 (maternal Indian heritage) to 1.15 (maternal Somalian heritage).

DISCUSSION

This study validates a gestational age prediction model in subgroups of infants born to immigrant mothers in Ontario, Canada. Our findings demonstrate that our previously published prediction model had excellent model performance characteristics in infants born to immigrants of diverse ethnicities/countries of origin, providing evidence that our modeling strategy will be robust for the estimation of gestational age in a variety of international settings.

Accurate estimation of gestational age is critical to guiding the course of newborn care. For women who do not access antenatal care through their local health care providers as a result of unrecognized pregnancy or inability to access obstetrical services as a result of social, financial or geographic impediments, reliable methods to determine the gestational age of their infants at birth would alleviate uncertainties surrounding delivery of appropriate newborn care for potentially premature infants.

For peer review only - http://bmjopen.bmj.com/site/about/guidelines.xhtml

Limitations of our approach include our reliance on surrogate measures of ethnicity, namely maternal country of birth rather than self-reported race or ethnicity, which was not available in the Canadian Landed Immigrant Database. Furthermore, naturalized immigrants living in North America are not likely to be representative of individuals living in the country of origin, either due to admixture (*i.e.* inter-racial families) or environmental factors (*e.g.* climate, socioeconomic status, diet, sanitation). Indeed, subtle variation in model performance in specific immigrant subgroups, such as those from Bangladesh and Somalia indicate a degree of biological or environmental influence that warrants consideration. While it is possible that prediction algorithms for postnatal determination of gestational age may be further refined by development of ethnicity-specific models, our results provide reassurance that our algorithm developed from an Ontario-based population performs consistently well across a range of ethnicities without modification.

The Bill and Melinda Gates Foundation seeks to provide equal access opportunities to healthy development and living. In line with the Foundation's strategic priorities to advance new approaches to measuring cognitive development and gestational age, refined prediction models capable of post-natal gestational age estimation across multi-ethnic populations are of high potential value in low-resource jurisdictions where access to antenatal ultrasound screening is limited. We are currently undertaking a series of international validation studies to compare the performance and feasibility of applying our model directly in infants born and living in targeted low-resource settings.

For peer review only - http://bmjopen.bmj.com/site/about/guidelines.xhtml

Funding Statement: This work was supported by The Bill and Melinda Gates Foundation Grand Challenges Exploration grant (OPP1141535). It was also supported by the Institute for Clinical Evaluative Sciences (ICES), which is funded by an annual grant from the Ontario Ministry of Health and Long-Term Care (MOHLTC).

Data Sharing Statement: All data used in this study were obtained from the Institute for Clinical Evaluative Sciences and are accessible to individuals with appropriate authorization

Competing Interests Statement: All authors have completed the ICMJE uniform disclosure form at www.icmje.org/coi_disclosure.pdf and declare: the authors had financial support from the Bill and Melinda Gates Foundation for the submitted work; no financial relationships with any organisations that might have an interest in the submitted work in the previous three years; no other relationships or activities that could appear to have influenced the submitted work.

The opinions, results and conclusions reported in this paper are those of the authors and are independent from the funding sources. No endorsement by ICES or the Ontario MOHLTC is intended or should be inferred. Parts of this material are based on data and information compiled and provided by the Canadian Institute of Health Information (CIHI). However, the analyses, conclusions, opinions and statements expressed herein are those of the author, and not necessarily those of CIHI.

For peer review only - http://bmjopen.bmj.com/site/about/guidelines.xhtml

REFERENCES

1 Woythaler MA, McCormick MC, Smith VC. Late preterm infants have worse 24-month neurodevelopmental outcomes than term infants. *Pediatrics*. 2011;127(3):e622-9.
2 Marlow N, Wolke D, Bracewell MA, et al. Neurologic and developmental disability at six years of age after extremely preterm birth. *N Engl J Med*. 2005;352(1):9-19.
3 Wilson K, Hawken S, Potter B, et al. Accurate prediction of gestational age using newborn screening analyte data. *Am J Obstet Gynecol*. 2016;214(4):513.e1-9.
4 Ryckman KK, Berberich SL, Dagle JM. Predicting gestational age using neonatal metabolic markers. *Am J Obstet Gynecol.* 2016;214(4):515.e1-.e13.
5 Jelliffe-Pawlowski LL, Norton ME, Baer RJ, et al. Gestational dating by metabolic profile at birth: a California cohort study. *Am J Obstet Gynecol*. 2016;214(4):511.e1-.e13.
6 Hanley JA, McNeil BJ. The meaning and use of the area under a receiver operating characteristic (ROC) curve. *Radiology*. 1982;143(1):29-36.

For peer review only

For peer review only - http://bmjopen.bmj.com/site/about/guidelines.xhtml

Author Contributions

SH and **RD** were involved in data acquisition and analysis. **SH, RD**, and **KW** were provided data interpretation. **SH, MM, KA,** and **KW** were involved in drafting and editing the manuscript. **KA** and **MM** provided project coordination. **BP** and **PC** provided critical edits to the manuscript for important intellectual content. **KW** was responsible for the conceptual design of the study.

For peer review only

For peer review only - http://bmjopen.bmj.com/site/about/guidelines.xhtml

Performance of a postnatal metabolic gestational age algorithm: a retrospective validation study among ethnic subgroups in Canada

Journal:	*BMJ Open*
Manuscript ID	bmjopen-2016-015615.R1
Article Type:	Research
Date Submitted by the Author:	08-May-2017

Complete List of Authors:	Hawken, Steven; Institute for Clinical Evaluative Sciences, ICES @uOttawa; The Ottawa Hospital Research Institute,<br>Ducharme, Robin; Institute for Clinical Evaluative Sciences<br>Murphy, Malia; Ottawa Hospital Research Institute<br>Atkinson, Katherine; Ottawa Hospital Research Institute<br>Potter, Beth; University of Ottawa, Epidemiology and Community Medicine<br>Chakraborty, Pranesh; Newborn Screening Ontario, Children's Hospital of Eastern Ontario<br>Wilson, Kumanan; Ottawa Hospital Research Institute, Clinical Epidemiology

**Primary Subject Heading**:	Epidemiology
Secondary Subject Heading:	Obstetrics and gynaecology
Keywords:	gestational age, prediction modelling, newborn screening

SCHOLARONE™
Manuscripts

For peer review only

Title: **Performance of a postnatal metabolic gestational age algorithm: a retrospective validation study among ethnic subgroups in Canada**

Authors: Steven Hawken[a,b,c], Robin Ducharme[c], Malia SQ Murphy[a], Katherine M Atkinson[a,d], Beth K Potter[a,b,c], Pranesh Chakraborty[e,f], Kumanan Wilson[a,b,c,g].

Affiliations: [a]Clinical Epidemiology Program, Ottawa Hospital Research Institute, Ottawa, Ontario, Canada
[b]School of Epidemiology, Public Health and Preventative Medicine, University of Ottawa, Ottawa, Ontario, Canada
[c]Institute for Clinical Evaluative Sciences (ICES uOttawa), Ottawa, Ontario, Canada
[d]Department of Public Health Sciences, Karolinska Institute, Stockholm, Sweden
[e]Department of Pediatrics, University of Ottawa, Ottawa, Ontario, Canada;
[f]Newborn Screening Ontario, Children's Hospital of Eastern Ontario, Ottawa, Ontario, Canada
[g]Department of Medicine, University of Ottawa, Ottawa, Ontario, Canada.

Corresponding Author: Kumanan Wilson, MD, MSc, FRCPC
Ottawa Hospital, Civic Campus
1053 Carling Avenue, Box 684
Administrative Services Building
Ottawa, ON K1Y 4E9
kwilson@ohri.ca
613-798-5555 ex 17921

Word Count: **1858**

For peer review only - http://bmjopen.bmj.com/site/about/guidelines.xhtml

ABSTRACT

Objectives: Biological modelling of routinely collected newborn screening data has emerged as a novel method for deriving postnatal gestational age estimates. Validation of published models have previously been limited to cohorts largely consisting of infants of white-Caucasian infant ethnicity. In this study, we sought to determine the validity of a published gestational age estimation algorithm among recent immigrants to Canada, where maternal landed immigrant status was used as a surrogate measure of infant ethnicity.

Design: We conducted a retrospective validation study in infants born in Ontario between April 2009 and September 2011.

Setting: Data were collected from the Institute for Clinical Evaluative Science at the Ottawa Hospital in Ontario, Canada.

Participants: The dataset included 230,034 infants born to non-landed immigrants and 70,098 infants born to immigrant mothers. The five most common countries of maternal origin were India (n=10,038), China (n=7,468), Pakistan (n=5,824), The Philippines (n=5,441), and Vietnam (n=1,408). Maternal country of origin was obtained from the Citizenship and Immigration Canada Landed Immigrant Database.

Primary and Secondary Outcome Measures: Performance of the postnatal gestational age algorithm was evaluated across non-immigrant and immigrant populations.

Results: RMSE of 1.05 weeks was observed for infants born to non-immigrant mothers, whereas RMSE ranged from 0.98–1.15 weeks amongst infants born to immigrant mothers. AUC for distinguishing term vs preterm infants (≥37 vs <37 weeks gestational age or >34 vs ≤34 weeks gestational age) were 0.958 and 0.986 respectively in the non-

For peer review only

immigrant subgroup and ranged from 0.927-0.964 and 0.966-0.99 in immigrant subgroups.

Conclusions: Algorithms for postnatal determination of gestational age may be further refined by development and validation of region or ethnicity-specific models; however, our results provide reassurance that an algorithm developed from Ontario-born infant cohorts performs well across a range of ethnicities and maternal countries of origin without modification.

Key Words: gestational age; prediction modelling; newborn screening

Strengths and Limitations of this Study

- This validation study has successfully demonstrated that a gestational age estimation algorithm performs well across infants from diverse backgrounds without modification.
- Population-Based Design: the validation cohort included 300,132 live-born infants born between April 2009 and September 2011 who underwent newborn screening at Newborn Screening Ontario.
- Defining Ethnic Subpopulations: Landed Immigrant status, rather than self-reported ethnicity was used as a surrogate measure for identification of ethnic subpopulations.
- Model Development: The model demonstrates poorer performance amongst more severely preterm infants, in part due to smaller numbers of preterm infants available for model development.

For peer review only - http://bmjopen.bmj.com/site/about/guidelines.xhtml

INTRODUCTION

Knowledge of gestational age at the time of birth is vital for ensuring adequate provision of newborn care, assessing neurocognitive function, and tracking of developmental milestones[1, 2]. For some pregnancies where antenatal ultrasound screening is not available, such as those occurring in jurisdictions with challenging socioeconomic conditions and/or limited access due to rurality, determination of gestational age can be challenging. Alternative antenatal dating methods, including last menstrual period and fundal height measurements, are hampered by poor recall history and a high prevalence of growth restriction, while commonly used postnatal assessments that score infants on neurologic and physical criteria are only accurate to within three to four weeks of true, ultrasound-validated gestational age[3]. World health and philanthropic organizations are now seeking novel ways of determining gestational age, both to improve individual care and to provide reliable, population-based estimates of preterm birth.

Secondary analysis of newborn screening samples, routinely collected within the first few days of an infant's life, has emerged as a unique opportunity for postnatal gestational age assessment. We and others have recently demonstrated the accuracy of postnatal gestational age algorithms derived from newborn screening data in three independent North American cohorts[4-6]. A significant limitation of the approaches published to date has been the predmoninance of white infants in the populations used for model validation. Metabolic profiles are subject to biological variation as a result of *in utero* environmental exposure[7], and recent work suggests that ethnic diversity within a population needs to be considered when establishing newborn screening reference

For peer review only - http://bmjopen.bmj.com/site/about/guidelines.xhtml

intervals for some conditions[8]. In light of this, this study sought to validate a postnatal gestational age metabolic model in ethnic subpopulations within Ontario, Canada.

MATERIALS AND METHODS

Study Design

We conducted a population-based retrospective validation study of infants born in the province of Ontario, using a combination of linked health administrative, newborn screening and immigration data maintained by the Institute for Clinical Evaluative Sciences (ICES). The Newborn Screening Ontario database includes the screening analyte profile of each infant who completed newborn screening (>99% of all infants born in Ontario). These screening analyte levels are used to screen for 29 conditions using a panel of over 40 screening analytes including acylcarnitines, amino acids, endocrine markers, enzymes and co-enzyme markers among others (Table 1). Maternal country of origin was used as a surrogate marker of infant ethnicity and was ascertained from the Citizenship and Immigration Canada Landed Immigrant Database. This study was approved by the Ottawa Health Science Network Research Ethics Board, Ottawa, Canada (20140724-01H).

Table 1. Newborn Screening Analytes used in model development

**Acylcarnitines**	C0, C2, C3, C3DC, C4, C4DC, C4OH, C5, C5:1, C5OH, C5DC, C6, C6DC, C8, C8:1, C10, C10:1, C12, C12:1, C14, C14:1, C14:2, C16, C16OH, C16:1OH, C18, C18:1, C18:2, C18OH, C18:1OH,
**Amino Acids**	alanine, arginine, citruline, glycine, leucine, methionine, ornithine, phenylalanine, tyrosine, valine,
**Endocrine Markers**	17-hydroxyprogesterone, thyroid stimulating hormone
**Enzyme and Co-Enzyme Markers**	galactose-1 phosphate uridyltransferase, biotinidase

For peer review only - http://bmjopen.bmj.com/site/about/guidelines.xhtml

Original Model

The postnatal gestational age estimation model previously published by our group included 249, 700 infants who were born between April 2007 and March 2009. The details of the original model have been described previously.[9] Briefly, infants who were identified as positive for any disorder screened for by newborn screening Ontario were excluded, as were infants with missing or unsatisfactory samples, gestational age and birthweight. Missing data occurred for a very proportion of the individual analytes used in the prediction model (<1%). Because all a full panel of analyte data per infant was required to score new observations, only infants with complete records for all analytes and other covariates were included in this validation exercise. The infants excluded due to missing gestational age, analytes or other covariates constituted <5% of the cohort. Gestational age was based on best obstetrical estimate (last menstrual period, dating ultrasound, or a combination).

The data were randomly partitioned into model development (50%), validation (25%) and test (25%) subsamples. Multiple linear regression was performed in the model development set, in which all analyte main effects were included in the model, as well as birthweight and sex. For analytes and birthweight, squared and cubic terms were also included. A stepwise variable selection algorithm was then conducted and all pairwise interactions were considered for inclusion. The *Schwartz Bayesian Criterion*, which rewards improved model fit and penalizes model complexity, was used in variable selection. Once this process was complete, the mean square error for the fitted model at each step was calculated in the independent validation sample subset, and the model with the smallest mean square error was selected. This approach provides a high level of

For peer review only - http://bmjopen.bmj.com/site/about/guidelines.xhtml

protection from overfitting in the final model.[9] Parameter estimates for the fitted model were fixed, and used to score (*i.e.,* estimate gestational age) in the independent test sample subset (n=62,434), and model performance characteristics (r-square, mean square error (MSE), proportion with observed versus estimated gestational age within one week) were calculated using this data, which was not used in model development. The final regression model included a total of 311 parameter estimates, including main effects, quadratic and cubic effects plus interaction terms.

The deviation of each calculated gestational age from the true gestational age of each infant is the residual model error for that infant (in unit, weeks). The residual model error can be positive or negative depending on the direction of the difference. The MSE is the sum of each of those residual errors after squaring it (also rendering all values positive) for all infants. MSE is in the units of $(\text{weeks})^2$. Taking the square root of the MSE yielded an overall "average deviation" in unit, weeks. An RMSE of 1 may be interpreted as the regression model estimating gestational age within ±1 week on average.

Gestational age was then dichotomized to distinguish term or preterm infant subgroups. Model performance to correctly classify infants across two thresholds of preterm birth categories by logistic regression analysis was assessed (area under the receiver operator curve, sensitivity, specificity and positive predictive value). Preterm birth thresholds were <37 weeks gestational age, the clinical threshold for preterm birth, and ≤34 weeks gestational age, a threshold that represents the lower limit of the late preterm period. Infants born ≤34 weeks gestational age (early preterm and severe preterm infants) are at increased health risk compared to those born late preterm or at term. Estimated gestational age identified by multiple linear regression was used as a

For peer review only - http://bmjopen.bmj.com/site/about/guidelines.xhtml

continuous independent variable in logistic regressions to determine the probability of being born preterm.[9]

Validation Cohort

The validation cohort included 300,132 live-born infants born between April 2009 and September 2011 who underwent newborn screening at Newborn Screening Ontario. A total of 348,098 records were available for the prescribed study period. Records with missing or implausible (data entry error) data on birthweight, sex and gestational age were removed from the analysis. Infants who received a ‘positive’ flag for any one of the newborn screening conditions were excluded. Also excluded were those with missing newborn screening analyte values, or those for which the samples were collected before 24 hours and after 7 days of birth. Figure 1 summarizes the logic used to create the study cohort. The validation cohort was completely independent from the data used in the development of the original model.

Model Validation

The coefficients from the reference linear regression model were fixed, and used to score the validation cohort. Calculated gestational age was used as the independent variable in logistic regression models to estimate the dichotomous categories of prematurity, for which model performance characteristics including AUC and sensitivity, specificity and positive predictive value were calculated. Confidence intervals for AUC were also calculated, using the approach described previously by Hanley and McNeil[10]. SAS 9.4 (Cary, NC, USA) was used for all statistical analyses.

For peer review only

For peer review only - http://bmjopen.bmj.com/site/about/guidelines.xhtml

RESULTS

The validation dataset included 230,034 infants born to non-immigrant mothers and 70, 098 born to immigrant mothers. The five most common countries of maternal birth were India (n=10,038), China (n=7,468), Pakistan (n=5,824), the Philippines (n=5,441), and Vietnam (n=1,408). The most common countries from the African continent were Somalia (n=833) and Nigeria (n=800), respectively. Descriptive characteristics of the validation cohort, including those for the above named countries are provided in Table 2.

Overall model performance characteristics for non-immigrant populations and the top eight landed immigrant subgroups are presented in Table 3. Model performance characteristics from the previous validation cohort are provided for comparison. Absolute gestational age estimation was on average accurate to within 1 week in continuous linear regression models (RMSE~1 week). RMSE ranged from 0.98 weeks (maternal Indian heritage) to 1.15 weeks (maternal Somalian heritage).

Model performance amongst preterm infants is provided in Table 4. Among non-immigrants, our algorithm performed comparably to our original validation with areas under the receiver operator curve (AUC) for classifying infants as term versus preterm; ≥37 versus <37 weeks gestational age, AUC=0.958 and >34 versus ≤34 weeks gestational age, AUC=0.986.

For peer review only - http://bmjopen.bmj.com/site/about/guidelines.xhtml

For peer review only

Table 2. Cohort Characteristics

	Non-Immigrants	India	China	Pakistan	Philippines	Vietnam	Bangladesh	Somalia	Nigeria
Sample Size	230,034	10,038	7,468	5,824	5,441	1,408	1,182	833	800
Birthweight, g	3382.91 ±558.85	3,185.42 ±518.73	3,315.79 ±469.78	3,208.88 ±504.10	3,177.88 ±528.21	3,221.91 ±473.36	3,114.52 ±508.14	3,362.01 ±559.40	3,357.89 ±588.54
Sex									
Male (%)	118,317 (51.43)	5,267 (52.47)	3,860 (51.69)	3,050 (52.37)	2,827 (51.96)	722 (51.28)	598 (50.59)	436 (52.34)	411 (51.38)
Female (%)	111,717 (48.57)	4,771 (47.53)	3,608 (48.31)	2,774 (47.63)	2,614 (48.04)	686 (48.72)	584 (49.41)	397 (47.66)	389 (48.63)
Gestational Age	38.84±1.78	38.72±1.70	38.87±1.51	38.73±1.61	38.44±1.76	38.69±1.58	38.54±1.83	39.10±1.82	38.57±1.89
≥37 weeks	212989 (92.60)	9,357 (93.22)	7,106 (95.15)	5,447 (93.53)	4,990 (91.71)	1,322 (93.89)	1,097 (92.81)	775 (93.04)	744 (93)
32-<37	15320 (6.66)	610 (6.08)	328 (4.39)	343 (5.89)	405 (7.44)	79 (5.61)	71 (6.01)	53 (6.36)	47 (5.88)
28-<32	1312 (0.57)	50 (0.5)	29 (0.39)	26 (0.45)	35 (0.64)	5 (0.36)	9 (0.76)	3 (0.36)	4 (0.5)
<28	413 (0.18)	21 (0.21)	5 (0.07)	8 (0.14)	11 (0.20)	2 (0.14)	5 (0.42)	2 (0.24)	5 (0.63)
Multi-gestation Pregnancy, n(%)	7,819 (3.40)	320 (3.19)	171 (2.29)	140 (2.40)	97 (1.78)	20 (1.42)	19 (1.61)	25 (3.00)	34 (4.25)
Low birthweight (<2500g), n(%)	12,716 (5.52)	787 (7.84)	307 (4.12)	410 (7.04)	427 (7.84)	72 (5.12)	100 (8.47)	48 (5.76)	51 (6.38)

For peer review only

For peer review only - http://bmjopen.bmj.com/site/about/guidelines.xhtml

Table 3. Model Performance comparison for Original Validation Study and new validation in immigrant and non-immigrant subgroups.

Cohort	RMSE (wks)	Infants with GA correctly classified									
		Overall		≥37 weeks		32 - <37 weeks		28 - <32 weeks		<28 weeks	
		±1 wks (%)	±2 wks (%)	±1 wks (%)	±2 wks (%)	±1 wks (%)	±2 wks (%)	±1 wks (%)	±2 wks (%)	±1 wks (%)	±2 wks (%)
Original Validation	1.06	66.8	94.9	69.1	96.4	39.0	75.6	46.5	76.9	50.7	77.5
**Validation based on immigration status in IRCC**											
New Validation Cohort (overall)	1.04	67.1	95.0	69.2	96.4	40.1	76.3	48.6	78.5	48.2	79.0
Non-immigrants	1.05	67.0	94.9	69.1	96.4	39.8	76.2	48.4	78.43	49.39	79.18
All Immigrants	1.04	67.7	95.2	69.6	96.4	41.4	76.9	49.5	78.9	44.7	82.3
India	1.04	68.1	95.2	69.8	96.3	44.4	80.0	42.0	70.0	42.9	85.7
China	0.98	71	96.2	72.9	97.6	31.4	67.4	51.7	75.9	20.0	80.0
Pakistan	1.05	67.2	95.3	68.4	96.2	48.7	82.2	61.5	88.5	62.5	87.5
Philippines	1.05	66.5	94.7	69.1	96.6	34.8	70.9	57.1	91.4	45.4	100.0
Vietnam	1	70	95.2	72.4	96.7	34.2	70.9	40.0	100.0	0.0	100.0
Bangladesh	1.14	67.4	94.6	69.1	95.9	45.1	77.5	44.4	77.8	60.0	80.0
Somalia	1.15	62.1	93.6	64.6	95.7	26.4	64.2	33.3	66.7	50.0	100.0
Nigeria	1.08	65.1	94.6	66.7	95.7	75.0	80.8	75.0	75.0	40.0	80.0

RMSE, root mean squared error; wks, weeks; GA, gestational age; IRCC, Citizenship and Immigration Canada's Permanent Resident Database.

For peer review only

Table 4. Comparison of model performance for predicting preterm status

Cohort	<37 weeks gestational age			≤34 weeks gestational age		
	**AUC (95%CI)**	**PPV 80%**	**p-value[a]**	**AUC (95%CI)**	**PPV 80%**	**p-value[a]**
Original Validation	0.970 (0.966,0.974)	-	ref	0.991 (0.987,0.995)	80.9	ref
**Validation based on immigration status in IRCC**						
New Validation Cohort (overall)	0.957 (0.956,0.959)	56.1	<0.0001	0.981(0.979,0.983)	75.9	<0.0001
Non-immigrants	0.958 (0.956,0.961)	57.4	<0.0001	0.981(0.979,0.983)	75.7	0.021
All Immigrants	0.954 (0.95,0.958)	51.7	<0.0001	0.983 (0.978,0.987)	74.3	0.0063
India	0.947 (0.936,0.959)	49.3	0.00027	0.980 (0.968,0.993)	61.8	0.240
China	0.957 (0.943,0.972)	42.0	0.092	0.983 (0.966,1.00)	86.3	0.78
Pakistan	0.949 (0.933,0.964)	43.1	0.010	0.960 (0.936,0.985)	77.0	0.103
Philippines	0.958 (0.945,0.971)	57.9	0.082	0.992 (0.982,1.00)	69.4	0.505
Vietnam	0.949 (0.916,0.981)	48.9	0.209	0.991 (0.965,1.02)	88.2	0.840
Bangladesh	0.945 (0.911,0.979)	49.3	0.151	0.947 (0.888,1.00)	69.6	0.300
Somalia	0.927 (0.880,0.974)	30.9	0.072	0.974 (0.924,1.02)	83.3	1.000
Nigeria	0.964 (0.930,0.998)	51.7	0.733	0.965 (0.910,1.02)	100.0	0.369

GA, gestational age; AUC, area under the ROC curve; IRCC, Citizenship and Immigration Canada's Permanent Resident Database. [a]p-value for two-sided z-test for the difference between original and subgroup AUC.

For peer review only

For peer review only - http://bmjopen.bmj.com/site/about/guidelines.xhtml

The model was able to discriminate between dichotomous preterm birth categories of immigrant infants with excellent precision. For discrimination of ≥37 versus <37 weeks gestation, AUC ranged from a 0.927 amongst infants of maternal Somalian descent to 0.964 amongst infants of maternal Nigerian heritage. Similarly, the model was able to discriminate well between >34 weeks versus ≤34 weeks gestational age, with AUC ranging from 0.966 for Nigerian and Bangladeshi infants to 0.994 amongst Phillipino infants.

DISCUSSION

This study validates a postnatal gestational age estimation model in subgroups of infants born to immigrant mothers in Ontario, Canada. Examination of performance characteristics demonstrated reasonable performance of our previously published model to determine gestational age in infants born to immigrants of diverse ethnicities/countries of origin. These findings provide proof-of-principle that metabolic modeling strategies will be robust in a variety of international infant cohorts.

The strength of our approach lies in our ability to use population-based datasets to aggregate health and administrative data for validation of our model with ample sample size to evaluate performance metrics across infant subgroups. In lieu of self-reported race or ethnicity, which were unavailable for our analyses, use of the Canadian Landed Immigrant Database provided data on infants born to immigrant mothers from a diverse range of countries. Naturalized immigrants living in North America may not be representative of individuals living in the country of origin however, either due to admixture (*i.e.* inter-racial families) or environmental factors (*e.g.* climate,

For peer review only - http://bmjopen.bmj.com/site/about/guidelines.xhtml

socioeconomic status, diet, sanitation). Indeed, whether subtle variation in model performance in specific immigrant subgroups, such as those from Bangladesh and Somalia, indicate a degree of biological or environmental influence warrants further consideration.

As we work towards evaluating our model in other infant populations, we must consider existing limitations to the published model. First, our original approach included a relatively small sample size of preterm infants for model development, which resulted in decreased accuracy amongst the most severely preterm infants[4]. We are now working to refine our model to optimize performance amongst all gestational age categories. Second, currently published models are complex, and require data on a large number of analytes measured by mass spectrometry. Ultimately, simplification of models to reduce the number metabolic variables while maintaining model performance will be required to streamline the approach for potential application in the field. An ideal model would be useful with analytes that are valid immediately after birth, can be measured using simple assays, and can be determined from cord blood.[11].

In summary, our results provide reassurance that an algorithm developed from an Ontario-based population performs consistently well across a range of ethnicities. Further validation studies will be required to evaluate the performance of this and other models in infants born and living across a range of international settings.

For peer review only - http://bmjopen.bmj.com/site/about/guidelines.xhtml

Funding Statement: This work was supported by The Bill & Melinda Gates Foundation Grand Challenges Exploration grant [OPP1141535]. It was also supported by the Institute for Clinical Evaluative Sciences (ICES), which is funded by an annual grant from the Ontario Ministry of Health and Long-Term Care (MOHLTC).

Competing Interests Statement: All authors have completed the ICMJE uniform disclosure form at www.icmje.org/coi_disclosure.pdf and declare: the authors had financial support from the Bill and Melinda Gates Foundation for the submitted work; no financial relationships with any organisations that might have an interest in the submitted work in the previous three years; no other relationships or activities that could appear to have influenced the submitted work.

The opinions, results and conclusions reported in this paper are those of the authors and are independent from the funding sources. No endorsement by ICES or the Ontario MOHLTC is intended or should be inferred. Parts of this material are based on data and information compiled and provided by the Canadian Institute of Health Information (CIHI). However, the analyses, conclusions, opinions and statements expressed herein are those of the author, and not necessarily those of CIHI.

Author Contributions

SH and **RD** were involved in data acquisition and analysis. **SH, RD**, and **KW** provided data interpretation. **SH, MM, KA,** and **KW** were involved in drafting and editing the manuscript. **KA** and **MM** provided project coordination. **BP** and **PC** provided critical

For peer review only - http://bmjopen.bmj.com/site/about/guidelines.xhtml

edits to the manuscript for important intellectual content. **KW** was responsible for the conceptual design of the study.

Data Sharing: no additional data available

For peer review only

For peer review only - http://bmjopen.bmj.com/site/about/guidelines.xhtml

REFERENCES

1. Woythaler MA, Mccormick MC, Smith VC. Late Preterm Infants Have Worse 24-Month Neurodevelopmental Outcomes Than Term Infants. *Pediatrics*. 2011;127(3):e622-9.
2. Marlow N, Wolke D, Bracewell MA, et al. Neurologic and Developmental Disability at Six Years of Age after Extremely Preterm Birth. *N Engl J Med*. 2005;352(1):9-19.
3. Lee AC, Mullany LC, Ladhani K, et al. Validity of Newborn Clinical Assessment to Determine Gestational Age in Bangladesh. *Pediatrics*. 2016;138(1).
4. Wilson K, Hawken S, Potter BK, et al. Accurate Prediction of Gestational Age Using Newborn Screening Analyte Data. *Am J Obstet Gynecol*. 2016;214(4):513 e1-9.
5. Ryckman KK, Berberich SL, Dagle JM. Predicting Gestational Age Using Neonatal Metabolic Markers. *Am J Obstet Gynecol*. 2016;214(4):515 e1-13.
6. Jelliffe-Pawlowski LL, Norton ME, Baer RJ, et al. Gestational Dating by Metabolic Profile at Birth: A California Cohort Study. *Am J Obstet Gynecol*. 2016;214(4):511 e1-13.
7. Ryckman KK, Berberich SL, Shchelochkov OA, et al. Clinical and Environmental Influences on Metabolic Biomarkers Collected for Newborn Screening. *Clin Biochem*. 2013;46(1-2):133-8.
8. Peters C, Brooke I, Heales S, et al. Defining the Newborn Blood Spot Screening Reference Interval for Tsh: Impact of Ethnicity. *J Clin Endocrinol Metab*. 2016;101(9):3445-9.
9. Wilson K, Hawken S, Potter B, et al. Accurate Prediction of Gestational Age Using Newborn Screening Analyte Data. *American journal of obstetrics and gynecology*. 2015.
10. Hanley JA, Mcneil BJ. The Meaning and Use of the Area under a Receiver Operating Characteristic (Roc) Curve. *Radiology*. 1982;143(1):29-36.
11. Wilson K, Hawken S, Murphy MS, et al. Postnatal Prediction of Gestational Age Using Newborn Fetal Hemoglobin Levels. *EBioMedicine*. 2017;15:203-9.

For peer review only - http://bmjopen.bmj.com/site/about/guidelines.xhtml

Figure List

Figure 1. Cohort Creation Strategy

For peer review only

For peer review only - http://bmjopen.bmj.com/site/about/guidelines.xhtml

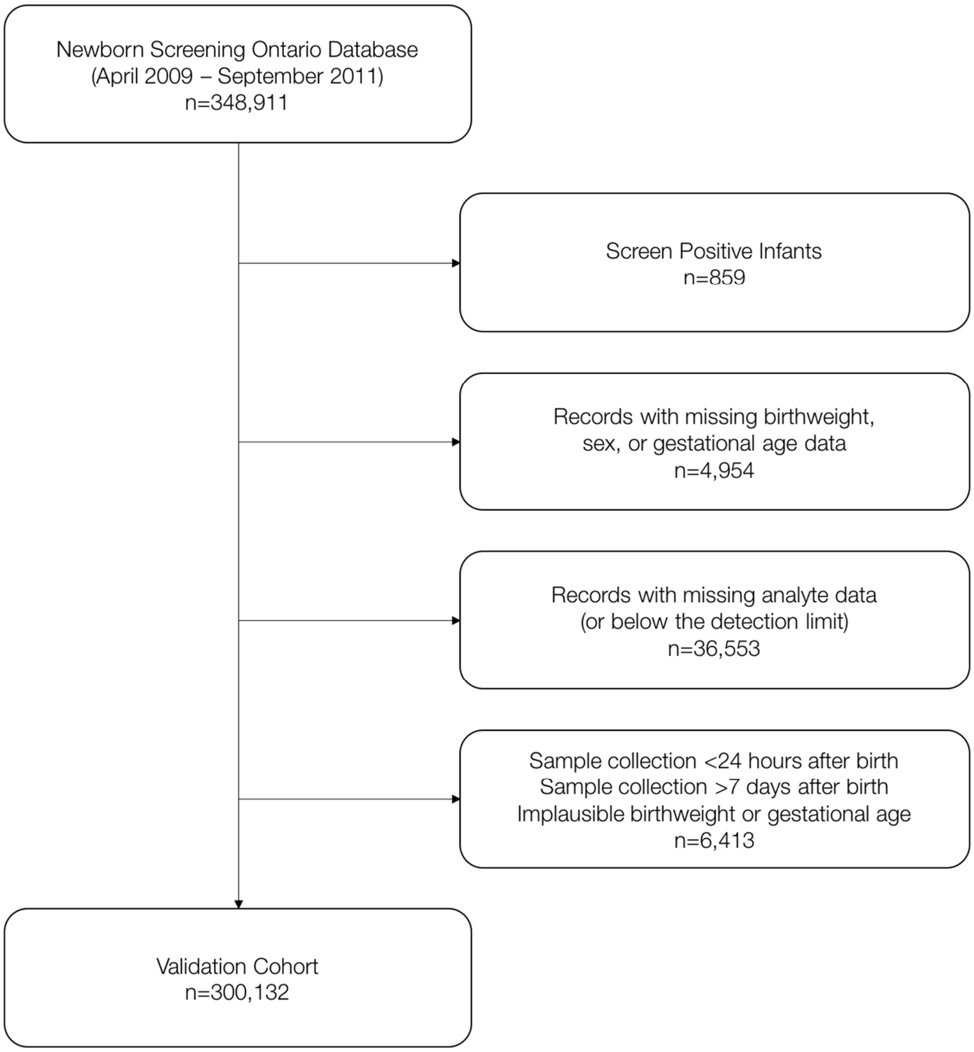

Cohort Creation Strategy

STROBE Statement—checklist of items that should be included in reports of observational studies

	Item No		Recommendation
**Title and abstract**	1	✓	(*a*) Indicate the study's design with a commonly used term in the title or the abstract
		✓	(*b*) Provide in the abstract an informative and balanced summary of what was done and what was found
**Introduction**			
Background/rationale	2	✓	Explain the scientific background and rationale for the investigation being reported
Objectives	3	✓	State specific objectives, including any prespecified hypotheses
**Methods**			
Study design	4	✓	Present key elements of study design early in the paper
Setting	5	✓	Describe the setting, locations, and relevant dates, including periods of recruitment, exposure, follow-up, and data collection
Participants	6	✓	(*a*) *Cohort study*—Give the eligibility criteria, and the sources and methods of selection of participants. Describe methods of follow-up<br>*Case-control study*—Give the eligibility criteria, and the sources and methods of case ascertainment and control selection. Give the rationale for the choice of cases and controls<br>*Cross-sectional study*—Give the eligibility criteria, and the sources and methods of selection of participants
			(*b*) *Cohort study*—For matched studies, give matching criteria and number of exposed and unexposed<br>*Case-control study*—For matched studies, give matching criteria and the number of controls per case
Variables	7	✓	Clearly define all outcomes, exposures, predictors, potential confounders, and effect modifiers. Give diagnostic criteria, if applicable
Data sources/ measurement	8*		For each variable of interest, give sources of data and details of methods of assessment (measurement). Describe comparability of assessment methods if there is more than one group
Bias	9		Describe any efforts to address potential sources of bias
Study size	10	✓	Explain how the study size was arrived at
Quantitative variables	11	✓	Explain how quantitative variables were handled in the analyses. If applicable, describe which groupings were chosen and why
Statistical methods	12	✓	(*a*) Describe all statistical methods, including those used to control for confounding
		✓	(*b*) Describe any methods used to examine subgroups and interactions
		✓	(*c*) Explain how missing data were addressed
			(*d*) *Cohort study*—If applicable, explain how loss to follow-up was addressed<br>*Case-control study*—If applicable, explain how matching of cases and controls was addressed<br>*Cross-sectional study*—If applicable, describe analytical methods taking account of sampling strategy
			(*e*) Describe any sensitivity analyses

Continued on next page

For peer review only

**Results**		
Participants	13* ✓	(a) Report numbers of individuals at each stage of study—eg numbers potentially eligible, examined for eligibility, confirmed eligible, included in the study, completing follow-up, and analysed
		(b) Give reasons for non-participation at each stage
	✓	(c) Consider use of a flow diagram
Descriptive data	14* ✓	(a) Give characteristics of study participants (eg demographic, clinical, social) and information on exposures and potential confounders
	✓	(b) Indicate number of participants with missing data for each variable of interest
		(c) *Cohort study*—Summarise follow-up time (eg, average and total amount)
Outcome data	15*	*Cohort study*—Report numbers of outcome events or summary measures over time
		Case-control study—Report numbers in each exposure category, or summary measures of exposure
		Cross-sectional study—Report numbers of outcome events or summary measures
Main results	16	(*a*) Give unadjusted estimates and, if applicable, confounder-adjusted estimates and their precision (eg, 95% confidence interval). Make clear which confounders were adjusted for and why they were included
	✓	(*b*) Report category boundaries when continuous variables were categorized
		(*c*) If relevant, consider translating estimates of relative risk into absolute risk for a meaningful time period
Other analyses	17	Report other analyses done—eg analyses of subgroups and interactions, and sensitivity analyses
**Discussion**		
Key results	18 ✓	Summarise key results with reference to study objectives
Limitations	19 ✓	Discuss limitations of the study, taking into account sources of potential bias or imprecision. Discuss both direction and magnitude of any potential bias
Interpretation	20 ✓	Give a cautious overall interpretation of results considering objectives, limitations, multiplicity of analyses, results from similar studies, and other relevant evidence
Generalisability	21 ✓	Discuss the generalisability (external validity) of the study results
**Other information**		
Funding	22 ✓	Give the source of funding and the role of the funders for the present study and, if applicable, for the original study on which the present article is based

*Give information separately for cases and controls in case-control studies and, if applicable, for exposed and unexposed groups in cohort and cross-sectional studies.

Note: An Explanation and Elaboration article discusses each checklist item and gives methodological background and published examples of transparent reporting. The STROBE checklist is best used in conjunction with this article (freely available on the Web sites of PLoS Medicine at http://www.plosmedicine.org/, Annals of Internal Medicine at http://www.annals.org/, and Epidemiology at http://www.epidem.com/). Information on the STROBE Initiative is available at www.strobe-statement.org.

For peer review only

BMJ Open

Performance of a postnatal metabolic gestational age algorithm: a retrospective validation study among ethnic subgroups in Canada

Journal:	*BMJ Open*
Manuscript ID	bmjopen-2016-015615.R2
Article Type:	Research
Date Submitted by the Author:	12-Jul-2017
Complete List of Authors:	Hawken, Steven; Institute for Clinical Evaluative Sciences, ICES @uOttawa; The Ottawa Hospital Research Institute,<br>Ducharme, Robin; Ottawa Hospital Research Institute, Clincial Epidemiology<br>Murphy, Malia; Ottawa Hospital Research Institute<br>Atkinson, Katherine; Ottawa Hospital Research Institute<br>Potter, Beth; University of Ottawa, Epidemiology and Community Medicine<br>Chakraborty, Pranesh; Newborn Screening Ontario, Children's Hospital of Eastern Ontario<br>Wilson, Kumanan; Ottawa Hospital Research Institute, Clinical Epidemiology
**Primary Subject Heading**:	Epidemiology
Secondary Subject Heading:	Obstetrics and gynaecology
Keywords:	gestational age, prediction modelling, newborn screening

SCHOLARONE™ Manuscripts

For peer review only

Title: **Performance of a postnatal metabolic gestational age algorithm: a retrospective validation study among ethnic subgroups in Canada**

Authors: Steven Hawken[a,b,c], Robin Ducharme[c], Malia SQ Murphy[a], Katherine M Atkinson[a,d], Beth K Potter[a,b,c], Pranesh Chakraborty[e,f], Kumanan Wilson[a,b,c,g].

Affiliations: [a]Clinical Epidemiology Program, Ottawa Hospital Research Institute, Ottawa, Ontario, Canada
[b]School of Epidemiology, Public Health and Preventative Medicine, University of Ottawa, Ottawa, Ontario, Canada
[c]Institute for Clinical Evaluative Sciences (ICES uOttawa), Ottawa, Ontario, Canada
[d]Department of Public Health Sciences, Karolinska Institute, Stockholm, Sweden
[e]Department of Pediatrics, University of Ottawa, Ottawa, Ontario, Canada;
[f]Newborn Screening Ontario, Children's Hospital of Eastern Ontario, Ottawa, Ontario, Canada
[g]Department of Medicine, University of Ottawa, Ottawa, Ontario, Canada.

Corresponding Author: Kumanan Wilson, MD, MSc, FRCPC
Ottawa Hospital, Civic Campus
1053 Carling Avenue, Box 684
Administrative Services Building
Ottawa, ON K1Y 4E9
kwilson@ohri.ca
613-798-5555 ex 17921

Word Count: **2007**

For peer review only

ABSTRACT

Objectives: Biological modelling of routinely collected newborn screening data has emerged as a novel method for deriving postnatal gestational age estimates. Validation of published models have previously been limited to cohorts largely consisting of infants of white-Caucasian infant ethnicity. In this study, we sought to determine the validity of a published gestational age estimation algorithm among recent immigrants to Canada, where maternal landed immigrant status was used as a surrogate measure of infant ethnicity.

Design: We conducted a retrospective validation study in infants born in Ontario between April 2009 and September 2011.

Setting: Data were collected from the Institute for Clinical Evaluative Science at the Ottawa Hospital in Ontario, Canada.

Participants: The dataset included 230,034 infants born to non-landed immigrants and 70,098 infants born to immigrant mothers. The five most common countries of maternal origin were India (n=10,038), China (n=7,468), Pakistan (n=5,824), The Philippines (n=5,441), and Vietnam (n=1,408). Maternal country of origin was obtained from the Citizenship and Immigration Canada Landed Immigrant Database.

Primary and Secondary Outcome Measures: Performance of the postnatal gestational age algorithm was evaluated across non-immigrant and immigrant populations.

Results: RMSE of 1.05 weeks was observed for infants born to non-immigrant mothers, whereas RMSE ranged from 0.98–1.15 weeks amongst infants born to immigrant mothers. AUC for distinguishing term vs preterm infants (≥37 vs <37 weeks gestational age or >34 vs ≤34 weeks gestational age) were 0.958 and 0.986 respectively in the non-

For peer review only - http://bmjopen.bmj.com/site/about/guidelines.xhtml

immigrant subgroup and ranged from 0.927-0.964 and 0.966-0.99 in immigrant subgroups.

Conclusions: Algorithms for postnatal determination of gestational age may be further refined by development and validation of region or ethnicity-specific models; however, our results provide reassurance that an algorithm developed from Ontario-born infant cohorts performs well across a range of ethnicities and maternal countries of origin without modification.

Key Words: gestational age; prediction modelling; newborn screening

Strengths and Limitations of this Study

- This validation study has successfully demonstrated that a gestational age estimation algorithm performs well across infants from diverse backgrounds without modification.
- Population-Based Design: the validation cohort included 300,132 live-born infants born between April 2009 and September 2011 who underwent newborn screening at Newborn Screening Ontario.
- Defining Ethnic Subpopulations: Landed Immigrant status, rather than self-reported ethnicity was used as a surrogate measure for identification of ethnic subpopulations.
- Model Development: The model demonstrates poorer performance amongst more severely preterm infants, in part due to smaller numbers of preterm infants available for model development.

For peer review only - http://bmjopen.bmj.com/site/about/guidelines.xhtml

INTRODUCTION

Knowledge of gestational age at the time of birth is vital not only for ensuring adequate provision of newborn care but also for assessing population level estimates of the burden of preterm birth to guide allocation of health services resources and targeted global health initiatives[1, 2]. In jurisdictions with challenging socioeconomic conditions and/or limited access to antenatal care (and thus ultrasound technology) due to rurality, determination of gestational age can be challenging. Other antenatal dating methods, including last menstrual period and fundal height measurements, are hampered by poor recall history and a high prevalence of growth restriction. Where prenatal estimations are unavailable or unreliable, a variety of standardized fetal assessments have been developed for clinicians seeking to determine fetal maturation after birth. Commonly used postnatal assessments that score infants on neurologic and physical criteria are only accurate to within three to four weeks of true, ultrasound-validated gestational age[3]. World health and philanthropic organizations are now seeking novel ways of determining gestational age at the time of birth, both to improve individual care and to provide reliable, high-quality data for population surveillance.

Secondary analysis of newborn screening samples, routinely collected within the first few days of an infant's life, has emerged as a unique opportunity for postnatal gestational age assessment. We and others have recently demonstrated the accuracy of postnatal gestational age algorithms derived from newborn screening data in three independent North American cohorts[4-6]. A significant limitation of the approaches published to date has been the predmoninance of white infants in the populations used for model validation. Metabolic profiles are subject to biological variation as a result of *in*

For peer review only - http://bmjopen.bmj.com/site/about/guidelines.xhtml

utero environmental exposure[7], and recent work suggests that ethnic diversity within a population needs to be considered when establishing newborn screening reference intervals for some conditions[8]. This study sought to validate a postnatal gestational age metabolic model in ethnic subpopulations within Ontario, Canada.

MATERIALS AND METHODS

Study Design

We conducted a population-based retrospective validation study of infants born in Ontario, Canada using a combination of linked health administrative, newborn screening and immigration datasets maintained by the Institute for Clinical Evaluative Sciences (ICES). The Newborn Screening Ontario (NSO) database includes the analyte profiles of each infant completing newborn screening in the province (>99% of all infants born in Ontario). Over 40 screening analytes and analyte ratios including acylcarnitines, amino acids, endocrine markers, enzymes and co-enzyme markers among others (Table 1) are available in the NSO database. Maternal country of origin was used as a surrogate marker of infant ethnicity and was ascertained from the Citizenship and Immigration Canada Landed Immigrant Database. This study was approved by the Ottawa Health Science Network Research Ethics Board, Ottawa, Canada (20140724-01H).

Table 1. Newborn Screening Analytes used in model development

**Acylcarnitines**	C0, C2, C3, C3DC, C4, C4DC, C4OH, C5, C5:1, C5OH, C5DC, C6, C6DC, C8, C8:1, C10, C10:1, C12, C12:1, C14, C14:1, C14:2, C16, C16OH, C16:1OH, C18, C18:1, C18:2, C18OH, C18:1OH,
**Amino Acids**	alanine, arginine, citruline, glycine, leucine, methionine, ornithine, phenylalanine, tyrosine, valine,
**Endocrine Markers**	17-hydroxyprogesterone, thyroid stimulating hormone

For peer review only - http://bmjopen.bmj.com/site/about/guidelines.xhtml

**Enzyme and Co-Enzyme Markers**	galactose-1 phosphate uridyltransferase, biotinidase

Original Model

The postnatal gestational age estimation model previously published by our group included 249, 700 infants born between April 2007 and March 2009. The details of the original model have been described previously.[9] Briefly, infants who were identified as positive for any disorder screened for by newborn screening Ontario were excluded, as were infants with unsatisfactory samples, and missing gestational age and birthweight. Because all a full panel of analyte data per infant was required to score new observations, only infants with complete records for all analytes and other covariates were included in this validation exercise. The infants excluded due to missing covariates including gestational age, analytes or others constituted <5% of the cohort. Gestational age was based on best obstetrical estimate (last menstrual period, dating ultrasound, or a combination). Note: >99% of women in Ontario receive at least one ultrasound during the course of pregnancy.[10]

The data were randomly partitioned into model development (50%), validation (25%) and test (25%) subsamples. Multiple linear regression was performed in the model development set, in which all analyte main effects were included in the model, as well as birthweight and sex. For analytes and birthweight, squared and cubic terms were also included. A stepwise variable selection algorithm was then conducted and all pairwise interactions were considered for inclusion. The *Schwartz Bayesian Criterion*, which rewards improved model fit and penalizes model complexity, was used in variable selection. Once this process was complete, the mean square error for the fitted model at

For peer review only - http://bmjopen.bmj.com/site/about/guidelines.xhtml

each step was calculated in the independent validation sample subset, and the model with the smallest mean square error was selected. This approach provides a high level of protection from overfitting in the final model.[9] Parameter estimates for the fitted model were fixed, and used to score (*i.e.,* estimate gestational age) in the independent test sample subset (n=62,434), and model performance characteristics (r-square, mean square error (MSE), proportion with observed versus estimated gestational age within one week) were calculated. The final regression model included a total of 311 parameter estimates, including main effects, quadratic and cubic effects plus interaction terms.

The deviation of each calculated gestational age from the true gestational age of each infant is the residual model error for that infant (in unit, weeks). The residual model error can be positive or negative depending on the direction of the difference. The MSE is the mean of each of those residual errors after squaring it (also rendering all values positive) for all infants. MSE is presented in the units of $(\text{weeks})^2$. Taking the square root of the MSE yielded an overall "average deviation" in unit, weeks.

Gestational age was then dichotomized to distinguish term or preterm infant subgroups. Model performance to correctly classify infants across two thresholds of preterm birth categories by logistic regression analysis was assessed (area under the receiver operator curve, sensitivity, specificity and positive predictive value). Preterm birth thresholds were <37 weeks gestational age, the clinical threshold for preterm birth, and ≤ 34 weeks gestational age, a threshold that represents the lower limit of the late preterm period. Infants born ≤ 34 weeks gestational age (early preterm and severe preterm infants) are at increased health risk compared to those born late preterm or at term. Estimated gestational age identified by multiple linear regression was used as a

For peer review only - http://bmjopen.bmj.com/site/about/guidelines.xhtml

continuous independent variable in logistic regressions to determine the probability of preterm birth.[9]

Validation Cohort

The validation cohort included 300,132 live-born infants born between April 2009 and September 2011 who underwent newborn screening at Newborn Screening Ontario. A total of 348,098 records were available for the prescribed study period. Records with missing or implausible (data entry error) data on birthweight, sex and gestational age were removed from the analysis. Infants who received a 'positive' flag for any one of the newborn screening conditions were excluded. Also excluded were those with missing newborn screening analyte values, or those for which the samples were collected before 24 hours and after 7 days of birth. Figure 1 summarizes the logic used to create the study cohort. The validation cohort was independent from the data used in the development of the original model.

Model Validation

The coefficients from the reference linear regression model were fixed, and used to score the validation cohort. Calculated gestational age was used as the independent variable in logistic regression models to estimate the dichotomous categories of preterm birth, for which model performance characteristics including AUC, sensitivity, specificity and positive predictive value were calculated. Confidence intervals for AUC were also calculated, using the approach described previously by Hanley and McNeil[11]. SAS 9.4 (Cary, NC, USA) was used for all statistical analyses.

For peer review only - http://bmjopen.bmj.com/site/about/guidelines.xhtml

RESULTS

The validation dataset included 230,034 infants born to non-immigrant mothers and 70, 098 born to immigrant mothers. The five most common countries of maternal birth were India (n=10,038), China (n=7,468), Pakistan (n=5,824), the Philippines (n=5,441), and Vietnam (n=1,408). The most common countries from the African continent were Somalia (n=833) and Nigeria (n=800), respectively. Descriptive characteristics of the validation cohort are provided in Table 2.

Overall model performance characteristics for non-immigrant populations and the top eight landed immigrant subgroups are presented in Table 3. Model performance characteristics from the original validation cohort are provided for comparison. Absolute gestational age estimation was to within 1 week in continuous linear regression models; RMSE ranged from 0.98 weeks (maternal Indian heritage) to 1.15 weeks (maternal Somalian heritage).

Model performance amongst preterm infants is provided in Table 4. Among non-immigrants, our algorithm performed comparably to our original validation with areas under the receiver operator curve (AUC) for classifying infants as term versus preterm; ≥37 versus <37 weeks gestational age, AUC=0.958 and >34 versus ≤34 weeks gestational age, AUC=0.986.

For peer review only - http://bmjopen.bmj.com/site/about/guidelines.xhtml

Table 2. Cohort Characteristics

	Non-Immigrants	India	China	Pakistan	Philippines	Vietnam	Bangladesh	Somalia	Nigeria
Sample Size	230,034	10,038	7,468	5,824	5,441	1,408	1,182	833	800
Birthweight, g	3382.91 ±558.85	3,185.42 ±518.73	3,315.79 ±469.78	3,208.88 ±504.10	3,177.88 ±528.21	3,221.91 ±473.36	3,114.52 ±508.14	3,362.01 ±559.40	3,357.89 ±588.54
Sex									
Male (%)	118,317 (51.43)	5,267 (52.47)	3,860 (51.69)	3,050 (52.37)	2,827 (51.96)	722 (51.28)	598 (50.59)	436 (52.34)	411 (51.38)
Female (%)	111,717 (48.57)	4,771 (47.53)	3,608 (48.31)	2,774 (47.63)	2,614 (48.04)	686 (48.72)	584 (49.41)	397 (47.66)	389 (48.63)
Gestational Age	38.84±1.78	38.72±1.70	38.87±1.51	38.73±1.61	38.44±1.76	38.69±1.58	38.54±1.83	39.10±1.82	38.57±1.89
≥37 weeks	212989 (92.60)	9,357 (93.22)	7,106 (95.15)	5,447 (93.53)	4,990 (91.71)	1,322 (93.89)	1,097 (92.81)	775 (93.04)	744 (93)
32-<37	15320 (6.66)	610 (6.08)	328 (4.39)	343 (5.89)	405 (7.44)	79 (5.61)	71 (6.01)	53 (6.36)	47 (5.88)
28-<32	1312 (0.57)	50 (0.5)	29 (0.39)	26 (0.45)	35 (0.64)	5 (0.36)	9 (0.76)	3 (0.36)	4 (0.5)
<28	413 (0.18)	21 (0.21)	5 (0.07)	8 (0.14)	11 (0.20)	2 (0.14)	5 (0.42)	2 (0.24)	5 (0.63)
Multi-gestation Pregnancy, n(%)	7,819 (3.40)	320 (3.19)	171 (2.29)	140 (2.40)	97 (1.78)	20 (1.42)	19 (1.61)	25 (3.00)	34 (4.25)
Low birthweight (<2500g), n(%)	12,716 (5.52)	787 (7.84)	307 (4.12)	410 (7.04)	427 (7.84)	72 (5.12)	100 (8.47)	48 (5.76)	51 (6.38)

For peer review only - http://bmjopen.bmj.com/site/about/guidelines.xhtml

Table 3. Model Performance comparison for Original Validation Study and new validation in immigrant and non-immigrant subgroups.

Cohort	RMSE (wks)	Infants with GA correctly classified									
		Overall		≥37 weeks		32 - <37 weeks		28 - <32 weeks		<28 weeks	
		±1 wks (%)	±2 wks (%)	±1 wks (%)	±2 wks (%)	±1 wks (%)	±2 wks (%)	±1 wks (%)	±2 wks (%)	±1 wks (%)	±2 wks (%)
Original Validation	1.06	66.8	94.9	69.1	96.4	39.0	75.6	46.5	76.9	50.7	77.5
**Validation based on immigration status in IRCC**											
New Validation Cohort (overall)	1.04	67.1	95.0	69.2	96.4	40.1	76.3	48.6	78.5	48.2	79.0
Non-immigrants	1.05	67.0	94.9	69.1	96.4	39.8	76.2	48.4	78.43	49.39	79.18
All Immigrants	1.04	67.7	95.2	69.6	96.4	41.4	76.9	49.5	78.9	44.7	82.3
India	1.04	68.1	95.2	69.8	96.3	44.4	80.0	42.0	70.0	42.9	85.7
China	0.98	71	96.2	72.9	97.6	31.4	67.4	51.7	75.9	20.0	80.0
Pakistan	1.05	67.2	95.3	68.4	96.2	48.7	82.2	61.5	88.5	62.5	87.5
Philippines	1.05	66.5	94.7	69.1	96.6	34.8	70.9	57.1	91.4	45.4	100.0
Vietnam	1	70	95.2	72.4	96.7	34.2	70.9	40.0	100.0	0.0	100.0
Bangladesh	1.14	67.4	94.6	69.1	95.9	45.1	77.5	44.4	77.8	60.0	80.0
Somalia	1.15	62.1	93.6	64.6	95.7	26.4	64.2	33.3	66.7	50.0	100.0
Nigeria	1.08	65.1	94.6	66.7	95.7	75.0	80.8	75.0	75.0	40.0	80.0

RMSE, root mean squared error; wks, weeks; GA, gestational age; IRCC, Citizenship and Immigration Canada's Permanent Resident Database.

For peer review only

For peer review only - http://bmjopen.bmj.com/site/about/guidelines.xhtml

Table 4. Comparison of model performance for predicting preterm status

Cohort	<37 weeks gestational age			≤34 weeks gestational age		
	**AUC (95%CI)**	**PPV 80%**	**p-value[a]**	**AUC (95%CI)**	**PPV 80%**	**p-value[a]**
Original Validation	0.970 (0.966,0.974)	-	ref	0.991 (0.987,0.995)	80.9	ref
**Validation based on immigration status in IRCC**						
New Validation Cohort (overall)	0.957 (0.956,0.959)	56.1	<0.0001	0.981(0.979,0.983)	75.9	<0.0001
Non-immigrants	0.958 (0.956,0.961)	57.4	<0.0001	0.981(0.979,0.983)	75.7	0.021
All Immigrants	0.954 (0.95,0.958)	51.7	<0.0001	0.983 (0.978,0.987)	74.3	0.0063
India	0.947 (0.936,0.959)	49.3	0.00027	0.980 (0.968,0.993)	61.8	0.240
China	0.957 (0.943,0.972)	42.0	0.092	0.983 (0.966,1.00)	86.3	0.78
Pakistan	0.949 (0.933,0.964)	43.1	0.010	0.960 (0.936,0.985)	77.0	0.103
Philippines	0.958 (0.945,0.971)	57.9	0.082	0.992 (0.982,1.00)	69.4	0.505
Vietnam	0.949 (0.916,0.981)	48.9	0.209	0.991 (0.965,1.02)	88.2	0.840
Bangladesh	0.945 (0.911,0.979)	49.3	0.151	0.947 (0.888,1.00)	69.6	0.300
Somalia	0.927 (0.880,0.974)	30.9	0.072	0.974 (0.924,1.02)	83.3	1.000
Nigeria	0.964 (0.930,0.998)	51.7	0.733	0.965 (0.910,1.02)	100.0	0.369

GA, gestational age; AUC, area under the ROC curve; IRCC, Citizenship and Immigration Canada's Permanent Resident Database. [a]p-value for two-sided z-test for the difference between original and subgroup AUC.

For peer review only

For peer review only - http://bmjopen.bmj.com/site/about/guidelines.xhtml

The gestational age estimation model was able to discriminate between dichotomous preterm birth categories of immigrant infants with robust precision. For discrimination of ≥37 versus <37 weeks gestation, AUC ranged from a 0.927 amongst infants of maternal Somalian descent to 0.964 amongst infants of maternal Nigerian heritage. Similarly, the model was able to discriminate well between >34 weeks versus ≤34 weeks gestational age, with AUC ranging from 0.966 for Nigerian and Bangladeshi infants to 0.994 amongst Phillipino infants.

For peer review only

DISCUSSION

This study validates a postnatal gestational age estimation model in subgroups of infants born to immigrant mothers living in Ontario, Canada. Examination of performance characteristics demonstrated reasonable performance of our previously published model to determine gestational age in infants born to immigrants of diverse countries of origin. These findings provide proof-of-principle that metabolic modeling strategies will be robust in a variety of international infant cohorts.

The strength of our approach lies in our ability to use population-based datasets to aggregate health and administrative data for validation of our model with ample sample size to evaluate performance metrics across infant subgroups. In lieu of self-reported race or ethnicity, which were unavailable for our analyses, use of the Canadian Landed Immigrant Database provided data on infants born to immigrant mothers from a diverse range of countries. We acknowledge however, that naturalized immigrants living in North America may not be representative of individuals living in the country of origin however, either due to admixture (*i.e.* inter-racial families) or environmental factors (*e.g.* climate,

For peer review only - http://bmjopen.bmj.com/site/about/guidelines.xhtml

socioeconomic status, diet, sanitation). Indeed, whether subtle variation in model performance in specific immigrant subgroups, such as those from Bangladesh and Somalia, indicate a degree of biological or environmental influence warrants further consideration.

As we work towards evaluating our model in other infant populations, we must consider existing limitations. First, our original approach included a relatively small sample size of preterm infants for model development, which is reflected in decreased accuracy of the model amongst the most severely preterm infants[4]. We are now working to refine our model to optimize performance amongst all gestational age categories. Overall precision of metabolic dating methods to within 1-2 weeks compares favourably to other commonly used postnatal dating methods, the accuracy of which vary widely (3-4 weeks)[3, 12, 13] depending on the method, level of training of the specialist performing the measurements and if the child is small for gestational age. Although limited sample size prevented evaluation of the model's amongst small-for gestational age or low-birthweight infants, recent work from our group has demonstrated comparable accuracy amongst this infant subpopulation[14]. This is particularly important when considering the potential to implement metabolic dating tools in LMIC given the prevalence of low birthweight infants in low-resource communities. In addition to continuous estimates, provision of gestational age estimates across dichotomous thresholds (eg ≥37 or <37 weeks gestational age) may be useful for the purpose of population surveillance. Second, currently published models are complex, and require data on a large number of analytes measured by mass spectrometry. Ultimately, simplification of models to reduce the number metabolic variables while maintaining model performance will be required to streamline

For peer review only - http://bmjopen.bmj.com/site/about/guidelines.xhtml

the approach for scalable, cost-effective, implementation. An ideal model would be useful with analytes that may be reliably measured from samples obtained immediately after birth (i.e cord blood), and are stable through weeks-months of appropriate storage prior to analysis.[14]

In summary, our results provide reassurance that an algorithm developed from an Ontario-based population performs consistently well across a range of ethnicities. Ultimately, further validation studies will be required to evaluate the performance of this and other postnatal dating models in infants born and living across a range of international settings to determine if a single global algorithm or multiple regional algorithms should be derived.

For peer review only

Funding Statement: This work was supported by The Bill & Melinda Gates Foundation Grand Challenges Exploration grant [OPP1141535]. It was also supported by the Institute for Clinical Evaluative Sciences (ICES), which is funded by an annual grant from the Ontario Ministry of Health and Long-Term Care (MOHLTC).

Competing Interests Statement: All authors have completed the ICMJE uniform disclosure form at www.icmje.org/coi_disclosure.pdf and declare: the authors had financial support from the Bill and Melinda Gates Foundation for the submitted work; no financial relationships with any organisations that might have an interest in the submitted work in the previous three years; no other relationships or activities that could appear to have influenced the submitted work.

The opinions, results and conclusions reported in this paper are those of the authors and are independent from the funding sources. No endorsement by ICES or the Ontario MOHLTC is intended or should be inferred. Parts of this material are based on data and information compiled and provided by the Canadian Institute of Health Information (CIHI). However, the analyses, conclusions, opinions and statements expressed herein are those of the author, and not necessarily those of CIHI.

Author Contributions

SH and **RD** were involved in data acquisition and analysis. **SH, RD**, and **KW** provided data interpretation. **SH, MM, KA,** and **KW** were involved in drafting and editing the manuscript. **KA** and **MM** provided project coordination. **BP** and **PC** provided critical

For peer review only - http://bmjopen.bmj.com/site/about/guidelines.xhtml

edits to the manuscript for important intellectual content. **KW** was responsible for the conceptual design of the study.

Data Sharing Statement

All data used in this study were obtained from the Institute for Clinical Evaluative Sciences and are accessible to individuals with appropriate authorization

For peer review only

For peer review only - http://bmjopen.bmj.com/site/about/guidelines.xhtml

REFERENCES

1. Woythaler MA, Mccormick MC, Smith VC. Late Preterm Infants Have Worse 24-Month Neurodevelopmental Outcomes Than Term Infants. *Pediatrics*. 2011;127(3):e622-9.
2. Marlow N, Wolke D, Bracewell MA, et al. Neurologic and Developmental Disability at Six Years of Age after Extremely Preterm Birth. *N Engl J Med*. 2005;352(1):9-19.
3. Lee AC, Mullany LC, Ladhani K, et al. Validity of Newborn Clinical Assessment to Determine Gestational Age in Bangladesh. *Pediatrics*. 2016;138(1).
4. Wilson K, Hawken S, Potter BK, et al. Accurate Prediction of Gestational Age Using Newborn Screening Analyte Data. *Am J Obstet Gynecol*. 2016;214(4):513 e1-9.
5. Ryckman KK, Berberich SL, Dagle JM. Predicting Gestational Age Using Neonatal Metabolic Markers. *Am J Obstet Gynecol*. 2016;214(4):515 e1-13.
6. Jelliffe-Pawlowski LL, Norton ME, Baer RJ, et al. Gestational Dating by Metabolic Profile at Birth: A California Cohort Study. *Am J Obstet Gynecol*. 2016;214(4):511 e1-13.
7. Ryckman KK, Berberich SL, Shchelochkov OA, et al. Clinical and Environmental Influences on Metabolic Biomarkers Collected for Newborn Screening. *Clin Biochem*. 2013;46(1-2):133-8.
8. Peters C, Brooke I, Heales S, et al. Defining the Newborn Blood Spot Screening Reference Interval for Tsh: Impact of Ethnicity. *J Clin Endocrinol Metab*. 2016;101(9):3445-9.
9. Wilson K, Hawken S, Potter B, et al. Accurate Prediction of Gestational Age Using Newborn Screening Analyte Data. *American journal of obstetrics and gynecology*. 2015.
10. Guttman A, Vermeulen M, Simeonov D, et al. Are Rates of Prenatal Ultrasound a Valid Measure of Health System Overuse? *Institute for Clinical Evaluative Sciences*. 2012.
11. Hanley JA, Mcneil BJ. The Meaning and Use of the Area under a Receiver Operating Characteristic (Roc) Curve. *Radiology*. 1982;143(1):29-36.
12. Spinnato JA, Sibai BM, Shaver DC, et al. Inaccuracy of Dubowitz Gestational Age in Low Birth Weight Infants. *Obstet Gynecol*. 1985;65(4):601-2.
13. Taylor RA, Denison FC, Beyai S, et al. The External Ballard Examination Does Not Accurately Assess the Gestational Age of Infants Born at Home in a Rural Community of the Gambia. *Ann Trop Paediatr*. 2010;30(3):197-204.
14. Wilson K, Hawken S, Murphy MS, et al. Postnatal Prediction of Gestational Age Using Newborn Fetal Hemoglobin Levels. *EBioMedicine*. 2017;15:203-9.

For peer review only - http://bmjopen.bmj.com/site/about/guidelines.xhtml

Figure List

Figure 1. Cohort Creation Strategy

For peer review only

For peer review only - http://bmjopen.bmj.com/site/about/guidelines.xhtml

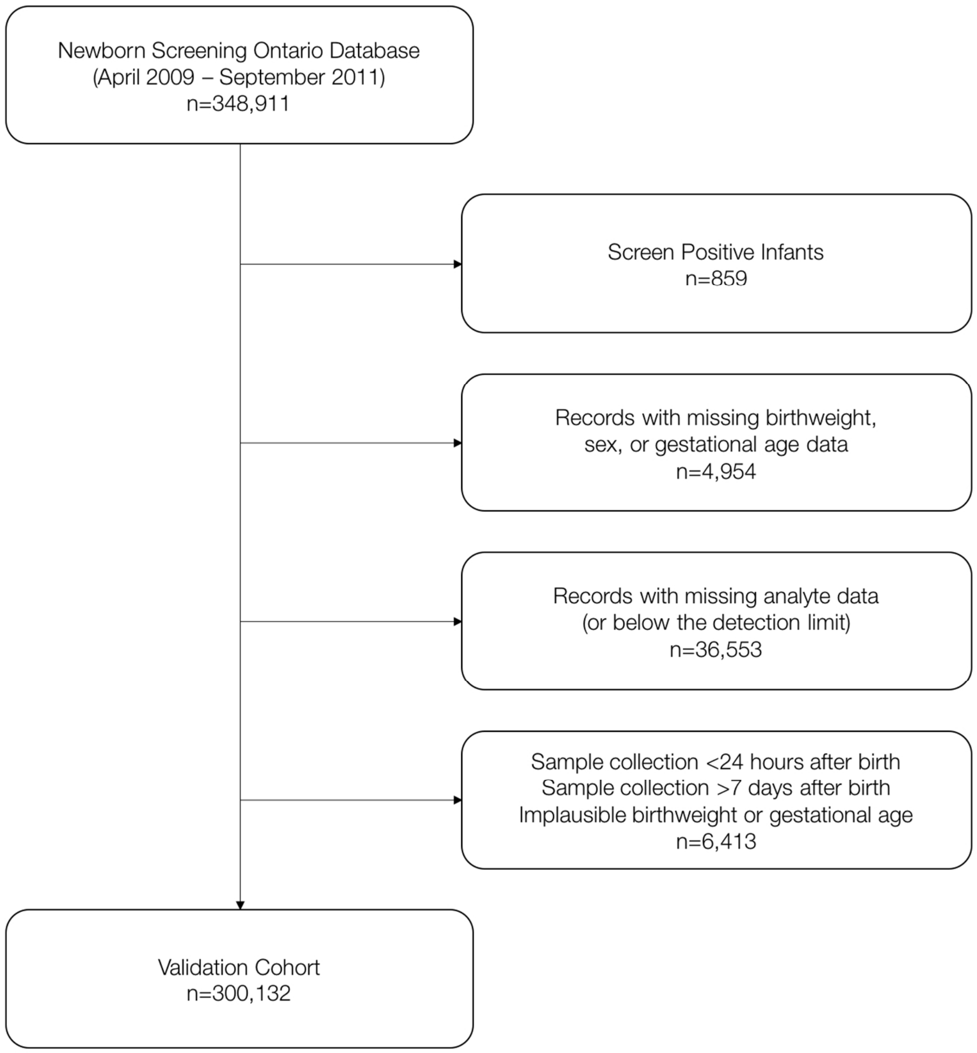

Cohort Creation Strategy

STROBE Statement—checklist of items that should be included in reports of observational studies

	Item No	Recommendation
**Title and abstract**	1 ✓	(*a*) Indicate the study's design with a commonly used term in the title or the abstract
	✓	(*b*) Provide in the abstract an informative and balanced summary of what was done and what was found
**Introduction**		
Background/rationale	2 ✓	Explain the scientific background and rationale for the investigation being reported
Objectives	3 ✓	State specific objectives, including any prespecified hypotheses
**Methods**		
Study design	4 ✓	Present key elements of study design early in the paper
Setting	5 ✓	Describe the setting, locations, and relevant dates, including periods of recruitment, exposure, follow-up, and data collection
Participants	6 ✓	(*a*) *Cohort study*—Give the eligibility criteria, and the sources and methods of selection of participants. Describe methods of follow-up<br>*Case-control study*—Give the eligibility criteria, and the sources and methods of case ascertainment and control selection. Give the rationale for the choice of cases and controls<br>*Cross-sectional study*—Give the eligibility criteria, and the sources and methods of selection of participants
		(*b*) *Cohort study*—For matched studies, give matching criteria and number of exposed and unexposed<br>*Case-control study*—For matched studies, give matching criteria and the number of controls per case
Variables	7 ✓	Clearly define all outcomes, exposures, predictors, potential confounders, and effect modifiers. Give diagnostic criteria, if applicable
Data sources/ measurement	8*	For each variable of interest, give sources of data and details of methods of assessment (measurement). Describe comparability of assessment methods if there is more than one group
Bias	9	Describe any efforts to address potential sources of bias
Study size	10 ✓	Explain how the study size was arrived at
Quantitative variables	11 ✓	Explain how quantitative variables were handled in the analyses. If applicable, describe which groupings were chosen and why
Statistical methods	12 ✓	(*a*) Describe all statistical methods, including those used to control for confounding
	✓	(*b*) Describe any methods used to examine subgroups and interactions
	✓	(*c*) Explain how missing data were addressed
		(*d*) *Cohort study*—If applicable, explain how loss to follow-up was addressed<br>*Case-control study*—If applicable, explain how matching of cases and controls was addressed<br>*Cross-sectional study*—If applicable, describe analytical methods taking account of sampling strategy
		(*e*) Describe any sensitivity analyses

Continued on next page

For peer review only - http://bmjopen.bmj.com/site/about/guidelines.xhtml

**Results**		
Participants	13* ✓	(a) Report numbers of individuals at each stage of study—eg numbers potentially eligible, examined for eligibility, confirmed eligible, included in the study, completing follow-up, and analysed
		(b) Give reasons for non-participation at each stage
	✓	(c) Consider use of a flow diagram
Descriptive data	14*✓	(a) Give characteristics of study participants (eg demographic, clinical, social) and information on exposures and potential confounders
	✓	(b) Indicate number of participants with missing data for each variable of interest
		(c) *Cohort study*—Summarise follow-up time (eg, average and total amount)
Outcome data	15*	*Cohort study*—Report numbers of outcome events or summary measures over time
		Case-control study—Report numbers in each exposure category, or summary measures of exposure
		Cross-sectional study—Report numbers of outcome events or summary measures
Main results	16	(*a*) Give unadjusted estimates and, if applicable, confounder-adjusted estimates and their precision (eg, 95% confidence interval). Make clear which confounders were adjusted for and why they were included
	✓	(*b*) Report category boundaries when continuous variables were categorized
		(*c*) If relevant, consider translating estimates of relative risk into absolute risk for a meaningful time period
Other analyses	17	Report other analyses done—eg analyses of subgroups and interactions, and sensitivity analyses
**Discussion**		
Key results	18 ✓	Summarise key results with reference to study objectives
Limitations	19 ✓	Discuss limitations of the study, taking into account sources of potential bias or imprecision. Discuss both direction and magnitude of any potential bias
Interpretation	20 ✓	Give a cautious overall interpretation of results considering objectives, limitations, multiplicity of analyses, results from similar studies, and other relevant evidence
Generalisability	21 ✓	Discuss the generalisability (external validity) of the study results
**Other information**		
Funding	22 ✓	Give the source of funding and the role of the funders for the present study and, if applicable, for the original study on which the present article is based

*Give information separately for cases and controls in case-control studies and, if applicable, for exposed and unexposed groups in cohort and cross-sectional studies.

Note: An Explanation and Elaboration article discusses each checklist item and gives methodological background and published examples of transparent reporting. The STROBE checklist is best used in conjunction with this article (freely available on the Web sites of PLoS Medicine at http://www.plosmedicine.org/, Annals of Internal Medicine at http://www.annals.org/, and Epidemiology at http://www.epidem.com/). Information on the STROBE Initiative is available at www.strobe-statement.org.

For peer review only
